# Case report: Misdiagnosed orolingual dyskinesia as a consequence of seizures in a chorea-acanthocytosis patient with a novel *VPS13A* variation from a family with consanguineous marriage

**DOI:** 10.3389/fneur.2024.1352467

**Published:** 2024-06-12

**Authors:** Mengying Wang, Huimin Li, Qing Zhou, Qin Zhao, Man Wang, Yumei Geng, Huicong Kang

**Affiliations:** ^1^Department of Neurology, Tongji Hospital, Tongji Medical College, Huazhong University of Science and Technology, Wuhan, Hubei, China; ^2^Department of Neurology, The Central Hospital of Wuhan, Wuhan, Hubei, China; ^3^Department of Neurology, Shanxi Bethune Hospital, Shanxi Academy of Medical Science, Tongji Shanxi Hospital, Third Hospital of Shanxi Medical University, Taiyuan, Shanxi, China; ^4^Key Laboratory of Vascular Aging, Ministry of Education, Tongji Hospital, Tongji Medical College, Huazhong University of Science and Technology, Wuhan, Hubei, China; ^5^Hubei Key Laboratory of Neural Injury and Functional Reconstruction, Huazhong University of Science and Technology, Wuhan, Hubei, China

**Keywords:** chorea-acanthocytosis, epilepsy, *VPS13A*, orolingual dyskinesia, deep brain stimulation

## Abstract

Chorea-acanthocytosis (ChAc) is a rare autosomal recessive inherited syndrome with heterogeneous symptoms, which makes it a challenge for early diagnosis. The mutation of *VPS13A* is considered intimately related to the pathogenesis of ChAc. To date, diverse mutation patterns of *VPS13A*, consisting of missense, nonsense, and frameshift mutations, have been reported. In this study, we first report a clinical case that was misdiagnosed as epilepsy due to recurrent seizures accompanied by tongue bite for 9 months, which was not rectified until seizures were controlled and involuntary orolingual movements with awareness became prominent and were confirmed to be orolingual dyskinesia. The patient was eventually diagnosed as ChAc based on whole-exome sequencing revealing novel homozygous c.2061dup (frameshift mutation) and c.6796A > T dual mutations in *VPS13A*. The patient from a family with consanguineous marriage manifested epileptic seizures at onset, including both generalized tonic–clonic seizures and absence but normal long-term electroencephalography, and gradually developed orofacial dyskinesia, including involuntary tongue protrusion, tongue biting and ulcers, involuntary open jaws, occasionally frequent eye blinks, and head swings. The first test of the peripheral blood smear was negative, and repeated checks confirmed an elevated percentage of acanthocytes by 15–21.3%. Structural brain MRI indicated a mildly swollen left hippocampus and parahippocampal gyrus and a progressively decreased volume of the bilateral hippocampus 1 year later, along with atrophy of the head of the caudate nucleus but no progression in 1 year. We deeply analyzed the reasons for long-term misdiagnosis in an effort to achieve a more comprehensive understanding of ChAc, thus facilitating early diagnosis and treatment in future clinical practice.

## 1 Introduction

Neuroacanthocytosis (NA) syndromes refer to a series of neurological abnormalities accompanied by the presence of acanthocytes on peripheral blood smears, which are composed of four core disorders, including chorea-acanthocytosis (ChAc), McLeod syndrome, pantothenate kinase-associated neurodegeneration (PKAN), and Huntington’s disease-like 2 (HDL2) (1). As a rare autosomal recessive neurodegenerative disease, ChAc is typically characterized by a choreatic movement disorder, psychiatric abnormalities, cognitive impairment, and often mild neuromuscular involvement (2).

The onset of ChAc was reported generally in young adulthood at the average age of 29 years old, with a life expectancy of approximately 11 years after diagnosis and an estimated suicidality rate of 10% (3). The mutation of the vacuolar protein sorting-associated protein 13A (*VPS13A*) gene has been proven to be intimately related to the pathogenesis of ChAc (4), with multiple mutation patterns, including missense (5), nonsense (6, 7), and frameshift (5, 7, 8). Early diagnosis is difficult based on the heterogeneous symptoms at onset, including chorea, orofacial dyskinesia, dystonia, Parkinsonism, and sometimes less specific symptoms such as epileptic seizures, psychiatric abnormalities, and neuropathy (5). Most typically, symptoms during the disease course are progressive abnormalities in movement (usually ataxia or hyperkinetic movements) with neurocognitive decline and behavioral changes (9). An elevated percentage of acanthocytes identified by peripheral blood smear, usually among 7–50%, is considered to be an important diagnostic biomarker (2, 4). Mildly elevated serum creatine kinase (CK) is also suggestive of the diagnosis (2). Typical neuroimaging alterations include atrophy of the head of the caudate nucleus on magnetic resonance imaging (MRI) and decreased metabolism of the caudate nucleus and putamen on 18F-fluorodeoxyglucose positron emission tomography (18F-FDG PET) (1).

Seizures are reported in 42% of ChAc patients at some point in the disease course and can occur early after the onset of involuntary movements or in the late stage of the disease course, but they can also be the predominant symptom and precede other clinical manifestations up to 15 years, which often cause delayed diagnosis (10–12). The most commonly reported type was generalized tonic–clonic seizure (GTCS) with generalized onset or secondary to focal onset, and focal seizure onset from the mesial temporal lobe has also been reported in a ChAc patient with hippocampus sclerosis (12, 13). The temporal lobe origin was also observed in another patient undergoing intracranial electroencephalography (EEG) for presurgical evaluation, who was subsequently diagnosed with ChAc (14). Seizures in ChAc patients are difficult to control at times and need constant adjustment on anti-seizure medications (ASMs) (10, 12). Worsened neurological conditions contributing to refractory seizures have also been reported (15). However, consideration of abnormal orofacial movements as side effects of ASMs and similar symptoms with Huntington’s disease (HD), Wilson disease, and Tourette syndrome, including dyskinesia, dystonia, choreographic movements, and tics, may cause mistaken or delayed diagnosis in clinical practice (10, 16).

In this study, we report a ChAc case with a novel homozygous *VPS13A* frameshift mutation. His onset manifested as recurrent tonic–clonic and absence seizures combined with tongue bites, and he was subsequently misdiagnosed as having epilepsy for 9 months. Not until the tongue bite without impaired consciousness appeared to be unresponsive to ASMs and orolingual dyskinesia was particularly noticed was the diagnosis of ChAc suspected and finally confirmed by whole-exome sequencing (WES). Most interestingly, the proband came from a consanguineous family whose parents were cousins. Based on the standards and guidelines of the American College of Medical Genetics and Genomics (ACMG) and the Association for Molecular Pathology, the *VPS13A* mutation of our patient was defined to be pathogenic (17), which has never been reported. We described the clinical characteristics in detail, compared with reported cases taking other mutation forms, and deeply discussed the reasons for long-term misdiagnosis in an effort to achieve a more comprehensive understanding of this rare disease, thus facilitating early diagnosis in future clinical practice.

## 2 Case description

A 39-year-old man with a past medical history (PMH) of hyperuricemia, gallstones, and duodenal ulcer surgery 20 years ago, he suddenly presented due to twitching of his bilateral arms and legs, without any triggers or auras, and subsequently fell to the ground with stiff limbs, a pale complexion, upturned eyes, a tightly closed jaw and foaming at the mouth but unaccompanied by tongue biting. During the entire episode, which lasted for 5 min, he was unconscious and unresponsive to calls from others. He was checked in a nearby hospital, with normal brain computed tomography (CT) and MRI and “mild abnormalities” of routine EEG report, without any details. Residual lags in response were complained of by his kin on the day of the attack, and the patient recovered to almost normal status the next day, except for generalized physical pain. Ten days later, he was referred to our epilepsy center for further evaluation. The routine EEG indicating multiple slow waves without a concentrative region, along with his typical symptoms and a family history of epilepsy, motivated the doctor to make a suspected diagnosis of epilepsy, but the patient refused long-term video electroencephalogram (VEEG) and further examination for etiology. Considering recurrent seizures were definitely unacceptable, he was treated with magnesium valproate at 750 mg per day and remained seizure-free.

Three months later, he developed intermittent absence seizures without tongue biting, lasting for 2–3 s, at a frequency of twice a month. He gradually developed involuntary tongue protrusion to the left and biting, along with involuntary open jaws, occasionally frequent eye blinks, and head swings another month later. The situation did not draw his attention until involuntary tongue biting became progressively frequent and severe another 2 months later, which occurred over 10 times a day but without impaired awareness. The abnormal movement led to protracted ulcers of the anterior two-third of the tongue on the left side, negatively influenced eating, and decreased appetite. Finally, he experienced a weight loss of 18 kilograms within 2 months and returned to the clinic. Unfortunately, his attending doctor failed to differentiate oropharyngeal dyskinesia from recurrent seizures and focused on the ASM regimen adjustment (increased the dosage of magnesium valproate to 1,000 mg per day, combined with oxcarbazepine at a daily dose of 600 mg and clonazepam 0.5 mg per night). One month later, the biting of the tongue was not improved, with a frequency that is already difficult to measure. To seek better control and the etiology for his “refractory epileptic tongue biting”, he was hospitalized.

## 3 Diagnostic assessment

The patient was born into a consanguineous family, and his younger brother suffered from epilepsy for years. For the neurological exam, he was normal except for an ulcer of the anterior two-third of the tongue on the left and a bilateral decreased tendon reflex.

**Figure 1:**
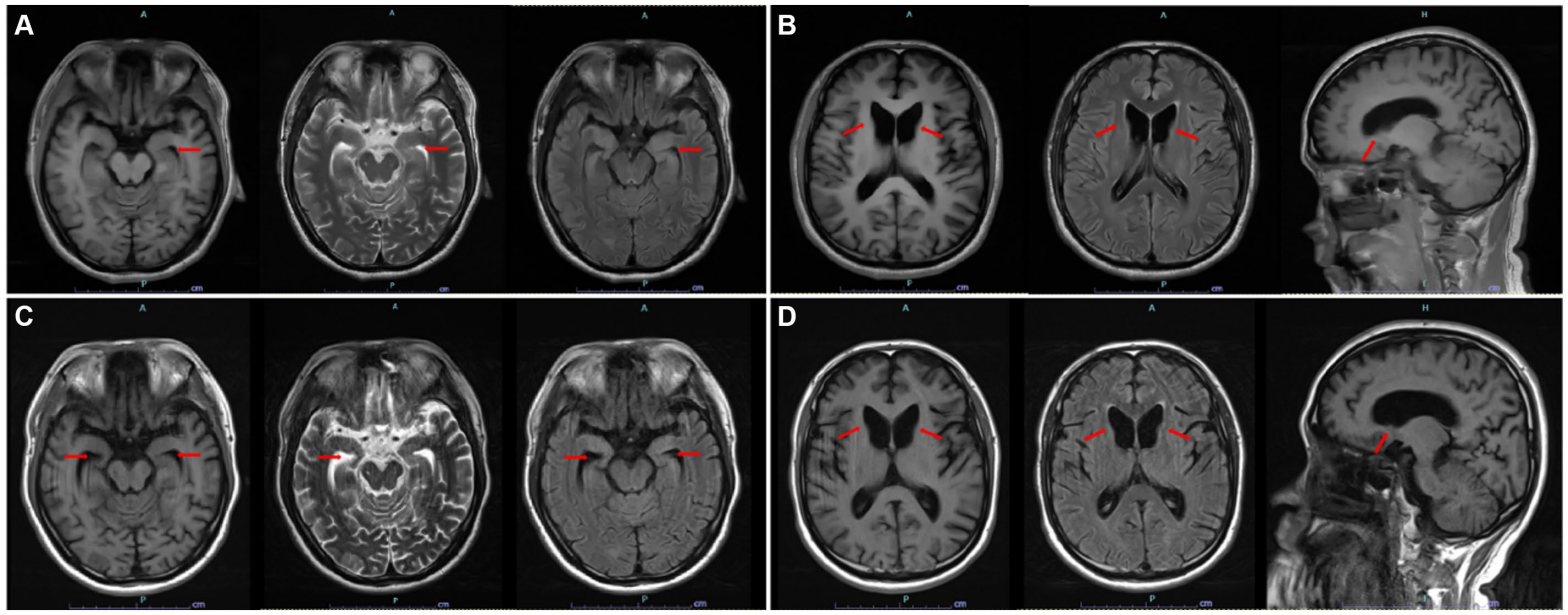
**(A)** Swollen left hippocampus and parahippocampal gyrus on axial T1, T2, and T2 FLAIR sequences and hyperintensity on T2 FLAIR sequences. **(B)** Atrophy of the head of the caudate nucleus and resulting enlarged anterior horn of the lateral ventricle on axial T1, T2Flair, and sagittal T1 sequences. **(C)** Decreased volume of the bilateral hippocampus and enlarged bilateral temporal horn of the lateral ventricles. **(D)** Almost the same degree of atrophy of the head of the caudate nucleus compared with last year. [**(A,B)** First structural brain MRI. **(C,D)** Reexamined structural brain MRI 1 year later. All images are marked by red arrows].

Serum levels of CK (307 U/L, normal range ≤ 190 U/L) and neuron-specific enolase (17.84 μg/L, normal range < 16.30 μg/L) were slightly elevated. The level of thyroid-stimulating hormone was reduced to 0.095 μIU/mL (normal range: 0.27–4.20 μIU/mL), and immunoglobulin M was reduced to 0.25 g/L (normal range: 0.46–3.04 g/L). Nevertheless, the majority of blood tests, including routine blood tests, coagulation function, erythrocyte sedimentation rate, serum copper, ceruloplasmin, lactate dehydrogenase, thyroid antibodies, anticardiolipin antibodies, antistreptolysin O and 14 other types of rheumatology-related antibodies, were all within the normal range. There were no apparent abnormalities in peripheral blood smears. Electrocardiogram and echocardiography were generally normal. Lumbar puncture was performed with normal routine, biochemical, and immune detection in the cerebrospinal fluid (CSF). Fifteen items of anti-neurocyte antibodies and 14 items of paraneoplastic antibodies were all negative both in the serum and CSF. Based on the suspicion of orolingual dyskinesia, which is typical for ChAc, the morphology examination of peripheral blood cells was performed and shown to be normal. Brain MRI indicated a mildly swollen left hippocampus and parahippocampal gyrus, appearing as slight hyperintensity on the T2 fluid-attenuated inversion recovery (FLAIR) sequence (Figure 1A), along with the atrophy of the head of the caudate nucleus resulting in an enlarged anterior horn of the lateral ventricle (Figure 1B). A 24-h VEEG was normal. Electromyography (EMG) of peripheral neuropathy revealed neurogenic damage in both the upper and lower limbs (involving motor sensory fibers, possibly combined with axonal and nerve root damage). The somatosensory evoked potential (SEP) demonstrated conduction dysfunction in the central segment of the bilateral somatosensory pathway, with a heavier lesion on the right side. To distinguish from the extrapyramidal symptoms attributed to HD or other inherited disorders, WES was further conducted, which can detect variations of exons and the surrounding introns in approximately 20,000 genes of the human genome using a targeted sequence capture method, and alignment to the human reference genome (hg19) was performed. The targeted sequences include known genetic disease-causing genes and those related to undetermined diseases, covering approximately 85% of known pathogenic variations. Haloperidol, aripiprazole, and tiapride hydrochloride were given to control involuntary perioral movement, with a slightly reduced frequency and severity of tongue biting. Given the relatively satisfactory control of seizures, the regimen of ASMs was unchanged with seizure-free. Therefore, the patient was discharged with the above oral drugs.

**Figure 2:**
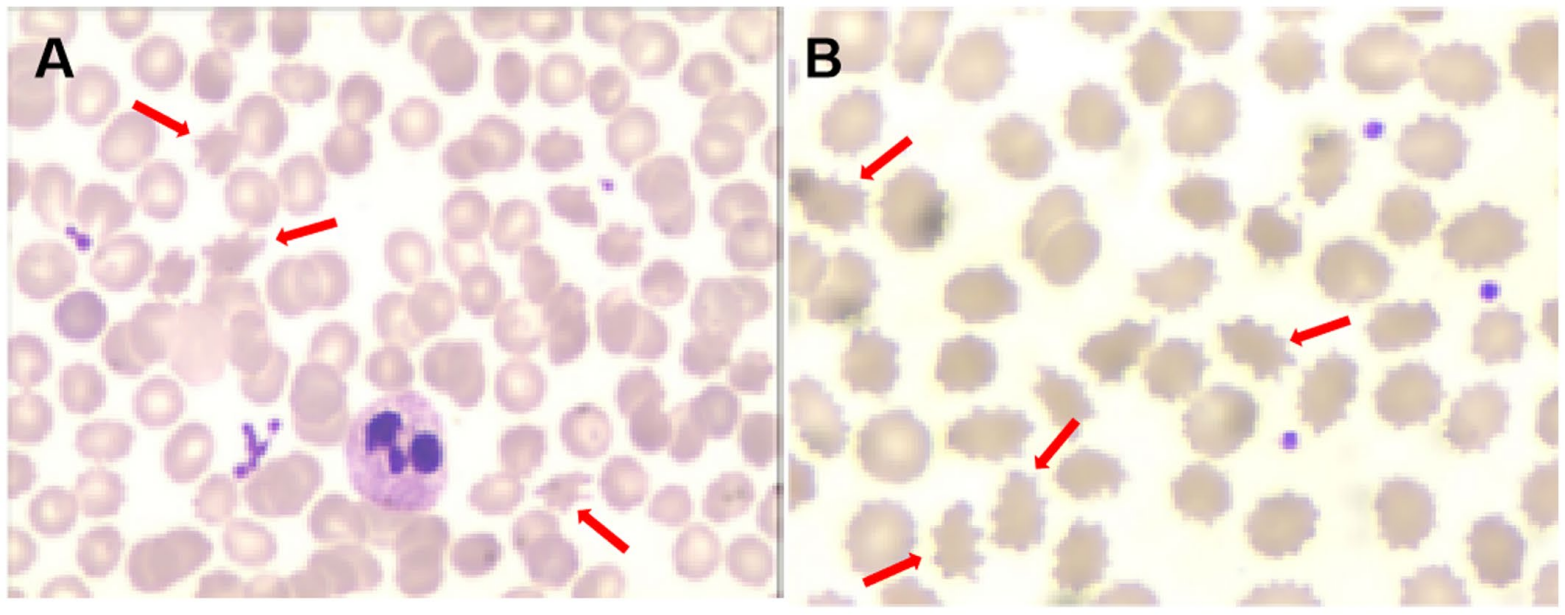
Acanthocytes on the second peripheral blood smear [**(A)**, red arrows] and the third peripheral blood smear [**(B)**, red arrows].

**Figure 3:**
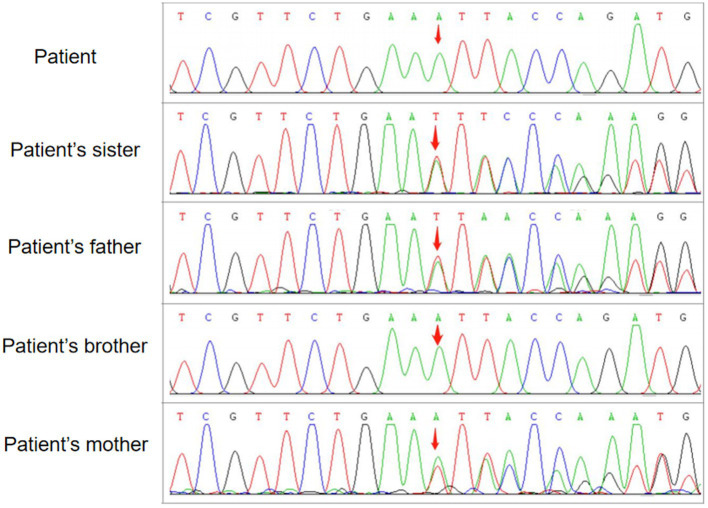
WES followed by confirmation by family verification, revealed the homozygous genotype of the patient and his brother and the heterozygous genotype of his parents and sister in the c.2061dup mutation of *VPS13A.*

**Figure 4:**
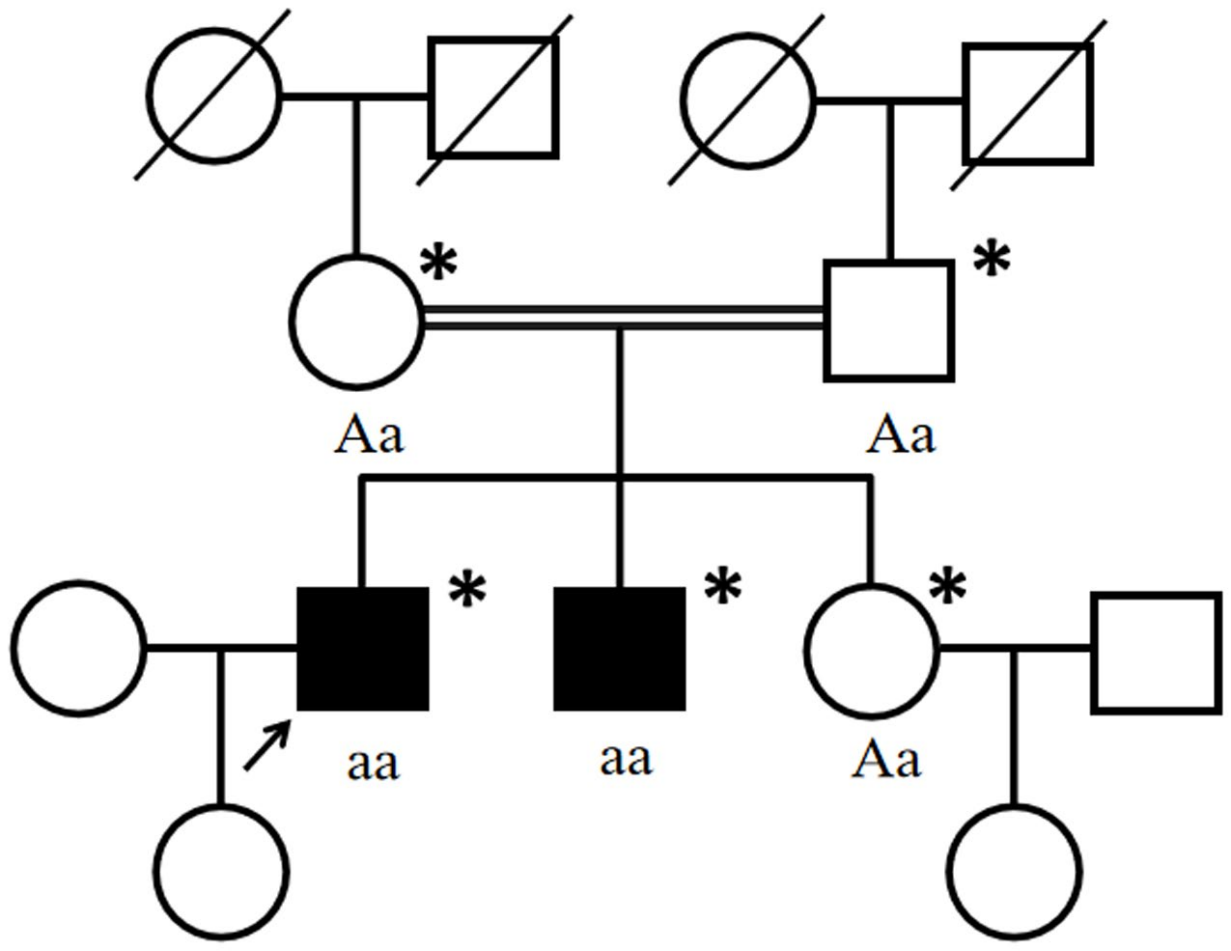
Pedigree chart of the affected family. Double lines indicate consanguineous unions. Square denotes a male family member, the circle represents a female family member, the slashed symbol indicates a deceased family member, the fully shaded symbol shows the patient with epilepsy, and the open symbol presents no symptoms. Arrow: index patient. Asterisk: genetic testing performed. Aa, heterozygous; aa, homozygous for c.2061dup (p. Leu688fs) in *VPS13A*.

One month later, WES indicated the homozygous c.2061dup and c.6796A > T mutations in *VPS13A*. Therefore, we performed a second peripheral blood smear and strongly emphasized the diagnosis of ChAc and the knack of gentle performance. Surprisingly but as expected, we discovered representative acanthocytes, and the proportion was over 15% (Figure 2A). Further family verification revealed a homozygous genotype of his brother (with a PMH of epilepsy) and a heterozygous genotype of his parents and sister (who were all asymptomatic) in the c.2061dup mutation of *VPS13A* (Figures 3, 4).

During the 12 months after the first hospitalization, he was seizure-free, and his involuntary movement was initially reversed for 3 months, although symptoms of orolingual hyperactivity fluctuated later. When he was readmitted for aggravated dyskinesia and tongue biting after catching two colds 1 year later, a repeated peripheral blood smear suggested an elevated proportion of acanthocytes (>20%) (Figure 2B). Blood biochemical examination indicated mild abnormalities of lipoprotein, with slightly elevated concentrations of triglycerides (TG, 2.11 mmol/L, normal range < 1.70 mmol/L), slightly reduced high-density lipoprotein levels (HDLs, 0.79 mmol/L, normal range 1.04–1.55 mmol/L), and normal levels of total cholesterol (TC, 3.53 mmol/L, normal range < 5.18 mmol/L) and low-density lipoprotein (LDL, 2.19 mmol/L, normal range < 3.37 mmol/L). Repeated MRI indicated a high possibility of hippocampal sclerosis based on decreased volume and increased signal on the T2 FLAIR sequence (Figure 1C). Nevertheless, no visual progression of atrophy of the head of the caudate nucleus was observed compared with the last year (Figure 1D). The patient was discharged with a slightly reduced frequency and degree of orolingual dyskinesia, including tongue protrusion, tongue biting and ulcers, involuntary open jaws, eye blinks, and head swing 10 days later. With seizure-free and normal VEEG, the dosage of magnesium valproate was gradually reduced to 500 mg per day. Three months later, due to the unsatisfactory therapeutic effect on orolingual dyskinesia, he was readmitted for presurgical evaluation for deep brain stimulation (DBS) in a specialized brain surgery hospital. EMG of tongue muscles revealed 4–4.8 Hz tremor potential on the left and 4.7–6.0 Hz on the right. No epileptiform discharges were captured on magnetoencephalogram (MEG). A fourth peripheral blood smear in another hospital indicated a proportion of 21.3% of acanthocytes, and regrettably, we failed to obtain the figures. The patient underwent DBS targeting the globus pallidus internus (GPi) with a stimulus voltage of 4 Hz, frequency of 130 Hz, and pulse width of 60 μs, showing well-controlled seizures under the ASM regimen as before and no tongue biting even after withdrawal of drugs on dystonia in our 1-year telephone follow-up.

## 4 Discussion

We report a case from a consanguineous family diagnosed with ChAc with a novel mutation in *VPS13A*, which primarily manifested as epileptic seizures and subsequently orolingual dyskinesia. Based on standards and guidelines of ACMG, the proband’s frameshift mutation (NM_033305: c.2061dup, p. Leu688fs) in *VPS13A* is pathogenic, given the following evidence: (1) This frameshift mutation may cause loss-of-function of encoded protein [pathogenic very strong (PVS) 1_Supporting]; (2) the frequency of this variation among population in the Genome Aggregation Database (gnomAD) is 0 [pathogenic moderate (PM) 2_Supporting]; (3) the mutation was homozygously detected in the proband, confirmed in *trans* (PM3_Supporting); and (4) cosegregation with the disease was found in the family [pathogenic supporting (PP) 1_Supporting]. After carefully retrieving, we found that the c.2061dup variant in *VPS13A* was neither documented in the gnomAD nor ClinVar databases. We further verified the pathogenicity of the detected mutation using Mutation Taster (mutationtaster.org) and the c.2061dup mutation in *VPS13A* was defined as “disease causing.”

In the ChAc cases that presented epilepsy as the first and prominent symptom, except for the homozygous frameshift mutation of c.2061dup in our case, nonsense mutations caused by c.8282C > G or c.4326 T > A, frameshift mutation caused by c.2343del or deletion of exons 70–73 and missense mutation caused by c.9263 T > G in exon 69 of *VPS13A* were reported, which all result in loss-of-function of the encoded protein around the C-terminus (6, 10, 12, 18, 19). As a gene spanning 240 kb and consisting of 73 exons, the C-terminus of *VPS13A* (amino acids 2,615–3,174) has been clearly demonstrated to be important for mitochondrial localization (19, 20). Whether the occurrence of seizures in ChAc is the consequence of disruption of mitochondrial localization needs further exploration.

We make further comparisons between these cases and ours in clinical manifestations, neuroimaging, and EEG features carefully to find several similarities and discrepancies. First, all the above-reported patients presented seizures as the initial symptoms, followed by other manifestations, including dysarthria, dysphagia, drooling, gait instability, and involuntary movements of limbs, trunk, orolingual, facial, or pharyngeal regions, except that the patient with the c.8282C > G mutation developed seizures preceded by mild non-coordinated movements for more than half a year. Second, in the matter of seizure types, GTCSs were reported in almost all the patients, except for one case with c.8282C>G mutation in *VPS13A*, whose detailed symptoms of seizures were not described, and one patient with deletion of exons 70–73, who manifested as auras of déjà-vu, fear, palpitations, and urinary urgency followed by loss of contact, regardless of primary or secondary. Consistent with the literature, our case developed one episode of GTCS as the initial symptom. Third, as an outstanding manifestation of our patient, orolingual involuntary movements were presented in the majority of previously reported cases, as well as those in facial and pharyngeal regions, while the cases with c.4326 T>A and c.8282C>G mutations only developed marginal non-coordinated movements or bradykinesia limited to limbs, accompanied by mild mandibular movements in the latter. Fourth, focusing on characteristics of neuroimaging, we found that in addition to caudate atrophy in most reported patients, a typical feature of ChAc, the patient with the c.8282C>G mutation presented slightly higher signals of bilateral hippocampus on T2 and T2 FLAIR sequences, and hippocampal atrophy was reported in two out of six cases with deletion of exons 70–73, which are, respectively, compliant with the neuroimaging features in the early and late stages in our case. This may indicate pathological abnormalities of the hippocampus and successive atrophy in ChAc patients presented with seizures. Fifth, in terms of EEG features, we surprisingly summarized abnormal cerebral activities located in unilateral or bilateral temporal regions in nearly all aforementioned cases, presented as epileptiform discharges or background slowing. However, generalized slowing and normal signals on EEG were, respectively, reported in three out of nine patients with the c.2343del mutation, and one of nine cases with deletion of exons 70–73 presented normal EEG as well. Our patient showed a generalized background slowing, consistent with one-third of reported cases with the c.2343del mutation. Finally, we found that a slight majority of nine patients with the c.2343del mutation showed an incomplete response to ASMs. Similarly, the patients with deletion of exons 70–73 showed poor responsiveness to ASMs or needed combination therapy in four out of the six patients. Differently, considerable responsiveness to ASMs was presented in patients carrying all the other mutation sites and our case, yet the response of seizures to ASMs needs to be proved in more patients.

His parents carried the heterozygous c.2061dup, given the autosomal recessive inheritance patterns of ChAc, which may explain their normal or unaffected state. Regrettably, until the follow-up, the patient’s brother, who carried the same homozygous genotype, did not report other symptoms of ChAc except for epileptic seizures. The phenotypic difference may be caused by the variation of individual genetic backgrounds and the impact of environmental factors as well. Additionally, there is still a possibility that the brother may develop the phenotype of our proband in the future, and we will keep following. Besides, we found that the c.6796A > T variant has been neither reported in previous literature nor documented in the gnomAD or ClinVar databases to be related to any diseases. Although compound mutations in *VPS13A* have been reported before (21), there was also no statement concerning the pathogenicity of the c.6796A > T variant. In summary, the simultaneous c.6796A > T mutation located behind c.2061dup was not considered to be the pathogenesis.

As the most common subtype of NA (22), ChAc commonly presents heterogeneous features, including orofacial dystonia, chorea, seizures, psychiatric symptoms, and rarely Parkinsonism (23). The initial and prominent symptom of our case is epileptic seizures, which can be attributed to countless causes, such as environmental, genetic, neurochemical, physiological, and structural origins in adults (24). A differential diagnosis of autoimmune encephalitis, paraneoplastic neurological syndrome, and some rheumatology-related diseases was conducted. The literature reveals that epileptic seizures occurring in ChAc are probably associated with decreased cortical GABAergic neurons, which are more prominent in the temporal lobe and may result in frequently reported seizures of temporal origin (25). Neuronal loss in the hippocampus and possible granule cell dispersion in the dentate gyrus, which were consistent with the criteria of hippocampal sclerosis, were found in a pathologic study on ChAc (13). GTCS is the most common type, and seizures can sometimes be drug-resistant (12, 18). Unfortunately, the exact proportion of patients with drug resistance was unclear because of limited studies. Patients with ChAc can also present as recurrent auras consisting of vertigo, déjà-vu, fear, hallucinations, palpitations, epigastric sensations, and dyscognitive seizures, such as unresponsiveness and oral automatisms, partly evolving into GTCS (10, 18, 26). Unilateral or bilateral epileptiform discharges in the temporal lobe on the EEG were reported (6, 10, 18). Similarly, but differently, our patient had only one episode of GTCS and paroxysmal absence seizures, which has not yet been reported in ChAc. The routine EEG performed at onset did not suggest a specific epileptogenic region, and VEEG carried out during hospitalization was normal, which may be due to the relatively deeper epileptogenic foci, restricted monitoring time, or the influence of ASM treatment for over 7 months. The slightly swollen volume and higher signal on T2 FLAIR of the left hippocampus and parahippocampal gyrus prompted a temporal-originated epileptogenic focus. A repeated MRI 1 year later indicated hippocampal sclerosis and further confirmed a potential temporal origin, which is consistent with previous reports (10, 13). The seizures in our case responded well to ASMs. The lack of progression of the atrophy degree of the head of the caudate nucleus probably suggested that pathological changes occurred early during the disease process, consistent with a previous study that revealed no significant correlation between disease duration and striatal volume or shape in ChAc patients (27). However, the progression of clinical symptoms and imaging in our patients still needs long-term follow-up.

Prominent orofacial features in ChAc patients include dysphagia, orofacial dyskinesia, traumatic bites, ulcers, and dysarthria (28). Orolingual dyskinesia was frequently reported as an early clinical symptom of ChAc (5), which gradually developed approximately 5 months after the epileptic episode (first symptom) in our case. Relevant symptoms included involuntary tongue protrusion and open jaws, frequent eye blinking, head swings, and tongue biting. Our case was misdiagnosed as poorly controlled epilepsy based on the shared characteristics of seizure attacks, including episodic, transient, repetitive, and stereotypical characteristics. Unfavorable therapeutic effects of the combined ASM regimen but increasingly prominent tongue biting with awareness led to the suspicion of extrapyramidal symptoms in neurodegenerative and gene-related disorders and confirmed orofacial dystonia. Interestingly, our patient showed an unsatisfactory response to anti-dystonia drugs but a superior effect on neuromodulation by GPi-DBS, which was demonstrated to be a promising treatment for chorea and dystonia in ChAc (29). In contrast to our patient, “improved” or “static” therapeutic effects were reported on involuntary movements in ChAc (5, 30).

During the diagnosis procedure, the first peripheral blood smear was negative, but the repeated two tests later showed an increasingly higher proportion of acanthocytes. Except for the natural development process of the disease, we analyzed the testing skill reason. First, a longer sample storage time may have an influence on the detection of acanthocytes. Before the first testing, the blood samples were stored for approximately half a day, which may reduce the positive rate. Second, careful and gentle operation skills are important. Careless and hasty performance may cause the targeted cells to fragment, consequently hampering the acquisition of precise results. Therefore, a negative result of the peripheral blood smear cannot rule out the diagnosis of ChAc, and repeated testing is necessary. Other results of critical accessory examinations, including mildly elevated serum CK and neurogenic damage of bilateral limbs involving motor and sensory fibers on the EMG, as well as decreased tendon reflex, are in line with the existing research findings (4).

## 5 Conclusion

We first reported a ChAc patient with a novel c.2061dup mutation in *VPS13A* from a consanguineous family in detail and performed a comprehensive literature review. We emphasize that for patients manifesting epileptic seizures and orolingual dystonia simultaneously, ChAc should be suspected, and careful clinical evaluation, including but not limited to peripheral blood smears, MRI, and genetic sequencing, is crucial for the diagnosis. Repeated testing of peripheral blood smears with immediate dispatch and gentle performance in the testing process is vitally important. Orolingual dystonia in ChAc patients may respond poorly to medications, and GPi-DBS may be a promising treatment. Our case expands our knowledge of the pathogenic mutation types of *VPS13A* and the phenotypes of ChAc. A comprehensive understanding of the disease and its key clinical diagnostic biomarkers for early diagnosis still needs clinical accumulation in the future.

## Data availability statement

The original contributions presented in the study are included in the article/supplementary material, further inquiries can be directed to the corresponding author.

## Ethics statement

The studies involving humans were approved by Ethics Committee on Human Research of Tongji Hospital. The studies were conducted in accordance with the local legislation and institutional requirements. The participants provided their written informed consent to participate in this study. Written informed consent was obtained from the individual(s) for the publication of any potentially identifiable images or data included in this article.

## Author contributions

MeW: Writing – original draft, Methodology, Formal analysis, Data curation. HL: Writing – original draft, Formal analysis, Data curation. QZho: Writing – original draft, Data curation, Methodology. QZha: Writing – original draft, Methodology. MaW: Writing – original draft, Formal analysis. YG: Writing – original draft, Methodology. HK: Writing – review & editing, Supervision, Project administration, Methodology, Funding acquisition, Conceptualization.
